# Neonatal Intensive Care Nurses’ Knowledge of Neonatal Pain Assessment in Private and Public Hospitals in Jeddah, Saudi Arabia: A Cross-Sectional Study

**DOI:** 10.7759/cureus.55189

**Published:** 2024-02-28

**Authors:** Ahmad Ismail

**Affiliations:** 1 Nursing, Fakeeh College for Medical Sciences, Jeddah, SAU

**Keywords:** saudi arabia, knowledge, nurses, nicu, neonates, pain assessment

## Abstract

Background: Neonatal Intensive Care Unit (NICU) nurses with adequate neonatal pain assessment knowledge are crucial in effective neonatal pain management. There is limited research that assessed the knowledge of NICU nurses in Saudi Arabia regarding neonatal pain assessment.

Objective: To assess the knowledge of NICU nurses in Saudi Arabia regarding neonatal pain assessment.

Design and methods: A cross-sectional design using an online survey was conducted to capture information regarding neonatal pain assessment knowledge from 125 NICU nurses in Saudi Arabia. Knowledge of pain assessment was assessed using a modified version of the knowledge, attitudes, and practice scale. Knowledge scores were classified as high, average, and low.

Results: Participants’ knowledge regarding neonatal pain assessment was inadequate (Mean = 63/100). The majority of the participants had a low to average level of knowledge (n= 97, 78%).

Conclusion: A significant proportion of NICU nurses had inadequate knowledge regarding neonatal pain assessment, which can be improved. Educational interventions are needed to boost these nurses' knowledge regarding neonatal pain assessment.

## Introduction

Adequate pain control depends on practical pain assessment [1-5]. Self-report of pain is considered the most legitimate method to capture the subjective experience of pain from patients with adequate capability [2,6-8]. Small babies nursed in the Neonatal Intensive Care Unit (NICU), however, cannot self-report their pain due to their language, cognitive development, and sickness limitations. Small babies experience many daily painful stimuli from required diagnostic and therapeutic procedures [9-14]. Such frequent exposure may lead to short and long-term negative significant consequences [9,13-16].

Nurses are responsible for pain assessment in many institutions [1]. In the NICU, nurses mainly use objective measures to assess for neonatal pain, such as physiological and behavioral changes [17-19]. Many evidence-based recommendations are available for nurses to use when they assess neonatal pain. It is instrumental for NICU nurses to adopt those neonatal pain assessment recommendations [1,19,20]. However, despite the availability of evidence-based recommendations for pain assessment, research still shows that neonatal pain assessment is not evidence-based and is suboptimal [1,21,22]. One important barrier to effective neonatal pain assessment is nurses' lack of knowledge regarding neonatal pain assessment [1]. Although some recent research reports that NICU nurses have gained more knowledge about neonatal pain assessment, there is still a gap between theory and practice [23]. Some NICU nurses do not even assess the level of pain based on validated scales developed for this purpose [1,24,25]. It is crucial to assess the knowledge of NICU nurses regarding neonatal pain assessment.

## Materials and methods

Study design

This research utilized a descriptive cross-sectional design to collect data regarding neonatal pain assessment knowledge from NICU nurses in Saudi Arabia. An online survey was conducted using the SurveyMonkey platform. Data were collected for six months between January and June 2023. This study is part of a project conducted in Saudi Arabia to improve the pain assessment and treatment of neonates in the NICU.

Study setting

The current online survey was shared with NICU nurses working in four hospitals in Jeddah, Saudi Arabia. The principal investigator shared the survey link and QR code with the unit managers, who shared them with the NICU nurses. These hospitals were private and public.

Sample and sampling

The number of NICU nurses in the four participating hospitals was 180. The sample of NICU nurses was calculated based on Slovin’s formula [26]. The required sample should follow $n = \frac{N}{1+Ne^{2}}$. Therefore, the minimum required sample is 122 NICU nurses. This study recruited 125 nurses, which is sufficient to represent the population of the four hospitals.

Data collection tools

The tool of this study included two sections: 1) demographic items and 2) the knowledge of pain assessment items. The demographic items included age, marital status, citizenship, education level, hospital type, and years of experience in neonatal nursing. A modified version of the tool based on the knowledge of NICU nurses on pain assessment among neonates was used. Permission from the original author [27] was obtained. The researcher modified the tool to include: 1) make it specific to the neonatal pain and pain assessment, 2) make it specific to knowledge, and 3) include items related to preterm and full-term neonates. The instrument was divided into two sections: 1) knowledge regarding neonatal pain (six items) and 2) knowledge regarding pain assessment (12 items). The following literature was further consulted to adapt the study tool: 1) Knowledge and Attitudes Survey regarding Pain by Ferrell et al., 2014 [28], 2) nurses' Knowledge and Attitude towards postoperative pain management in Ghana by Adams et al., 2020 [29], 3) pain assessment and management in neonatal intensive care units in the Eastern Province of Saudi Arabia by Alburaey et al., 2020 [24], 4) oral sucrose for neonatal pain: perception of Jordanian nurses by Kassab et al., 2021 [30], and 5) the knowledge and practice of nurses and associated factors in managing neonatal pain at selected public hospitals in Addis Ababa, Ethiopia survey by Wari et al., 2021 [31].

The data collection tool used in this study was developed in English, and no translation was conducted, considering that the sample consisted of educated nurses proficient in English. Further, the study tool was validated by a panel of six experts in nursing, NICU, pain, and research. The content validity index reached 89%. Internal consistency reliability was examined by Kuder-Richardson Formula 20, which showed a very good reliability value (KR20 =0.84).

Data collection procedure

Ethical approval was obtained from Fakeeh College for Medical Sciences (357/IRB/2022) and the Directorate of Health Affairs in Jeddah (A01613). After obtaining ethical approvals from these two local ethical committees, the principal investigators arranged an initial visit to the target settings and met with the NICU managers. The primary purpose of the study, expected outcomes, and clinical implications were introduced to the unit managers. The survey link and QR code were shared with the NICU managers, who distributed them to the NICU staff. The first page of the survey included information on the study and the consent to participate option. If a participant decided to participate, she clicked on the “agree to participate” button, where the platform moved her to the survey items. If the participant did not agree to participate, she clicked on the “disagree to participate” button, and then the platform thanked and exited her. Participants had the chance to seek further clarification about the study from the unit managers. The principal investigator has visited the units biweekly for follow-up and to answer any raised questions. The role of the principal investigator when visiting the site was to answer questions about the study to the unit managers who answered the participants. The principal investigator was aware of his role and did not meet or impact the participants when completing the survey.

Data analysis

The Statistical Package for Social Sciences (SPSS) software (IBM Corp., Armonk, NY, USA) was used to analyze the data. The collected data was exported from the SurveyMonkey platform to the SPSS. Data were coded, entered, and analyzed. Data were screened, and missing were checked. Descriptive statistics using mean and standard deviation were used to summarize the continuous variables. Frequencies and percentages were used to summarize the categorical variables. The total knowledge score of neonatal pain assessment was a sum of the correct answers divided by the number of items (18). Then, it was classified as high knowledge (80%-100%), average (60%-79%), and low (0-59%). Independent sample t-test and One-Way ANOVA were used to find the differences between the categorical groups (marital status, citizenship, education level, and hospital type) in nurses' knowledge of neonatal pain assessment. The Pearson correlation coefficient was used to test the relationship between the continuous variables (age and experience) and nurses' knowledge regarding neonatal pain assessment.

Ethical considerations

Completion and submission of the survey implied consent to participate. The study was voluntary and anonymous. Data was collected using the SurveyMonkey Platform, which has security measures in place to protect the confidentiality of the participant’s data. Participants' data will be destroyed by secured deletion five years after the publication of the results. Data is stored using a password-protected file. No one is allowed to access the data except the principal investigators. Data are stored at the principal investigator’s institution.

## Results

All participants were females (n = 125, 100%). The mean age of them was 32 years ± 8. The mean years of experience in neonatal nursing was 7 years ±5. The majority hold a bachelor’s degree in nursing (n = 99, 79%) and work for public hospitals (n= 95, 76%). Nearly half (n = 66, 53%) were non-Saudi (Table 1).

**Table 1:** Participants' demographics

Variable	Mean ± SD
Age	32 ± 8
Years of experience in neonatal nursing	7 ± 5
Marital status:	n (%)
Single	56 (45)
Married	69 (55)
Citizenship:	
Saudi	59 (47)
Non-Saudi	66 (53)
Education:	
College diploma	17 (14)
Bachelor	99 (79)
Master and above	9 (7)
Hospital Type:	
Public	95 (76)
Private	30 (24)

Participants’ average knowledge score of neonatal pain assessment was 63%. The highest number of correct answered items were “minor procedures can cause pain in neonates” (n = 119, 95%) and “neonatal pain should be assessed routinely (n = 104, 83%).” The lowest correctly answered items were “The cry, required oxygen, increased vital signs, expression, and sleeplessness scale (CRIES) is a validated assessment scale for pre-term neonates” (n = 19, 15%), “physiological indicators for pain, such as heart rate, are always valid to assess neonatal pain” (n = 40, 32%), “the premature infant pain profile (PIPP) is a validated pain scoring system for term neonates (n = 40, 32%), and “the standard numeric 0-10 pain scale is a validated scale for neonatal pain assessment” (n= 62, 50%) (Table 2).

**Table 2:** Knowledge of NICU nurses regarding neonatal pain assessment NICU= Neonatal Intensive Care Unit; T= True; F= False; CRIES= The cry, required oxygen, increased vital signs, expression, and sleeplessness scale; PIPP= The premature infant pain profile.

No	Correct Answer	Item	Number of correct answers	%
1	T	Neonates are capable of experiencing pain.	94	75
2	T	Minor procedures can cause pain.	119	95
3	T	Preterm neonates are at a greater risk of neurodevelopment impairment due to repeated painful procedures.	94	75
4	T	Neonates are more sensitive to pain than older children and adults.	92	74
5	T	Preterm neonates are more sensitive to pain stimuli than full-term infants.	78	62
6	T	Neonatal pain has long-term adverse effects.	78	62
7	F	Neonatal pain assessment should be done when the baby cries only.	93	74
8	T	Neonatal pain assessment should be done using a validated assessment tool.	92	74
9	T	The neonatal nurse uses behavioral indicators such as facial expressions to assess neonatal pain.	90	72
10	T	Neonatal nurses may use physiological indicators such as heart rate to assess neonatal pain.	91	73
11	F	Physiological indicators for pain, such as heart rate, are always valid to assess neonatal pain.	40	32
12	T	Neonatal pain should be assessed routinely.	104	83
13	F	The standard numeric 0–10 pain scale is a validated scale for neonatal pain assessment.	62	50
14	T	The CRIES is a validated assessment scale for term neonates.	89	71
15	T	The CRIES is a validated assessment scale for pre-term neonates.	19	15
16	T	The PIPP is a validated pain scoring system for preterm neonates.	70	56
17	T	The PIPP is a validated pain scoring system for term neonates.	40	32
18	F	There is no need to document the pain assessment.	82	66
		Mean		63

**Figure 1:**
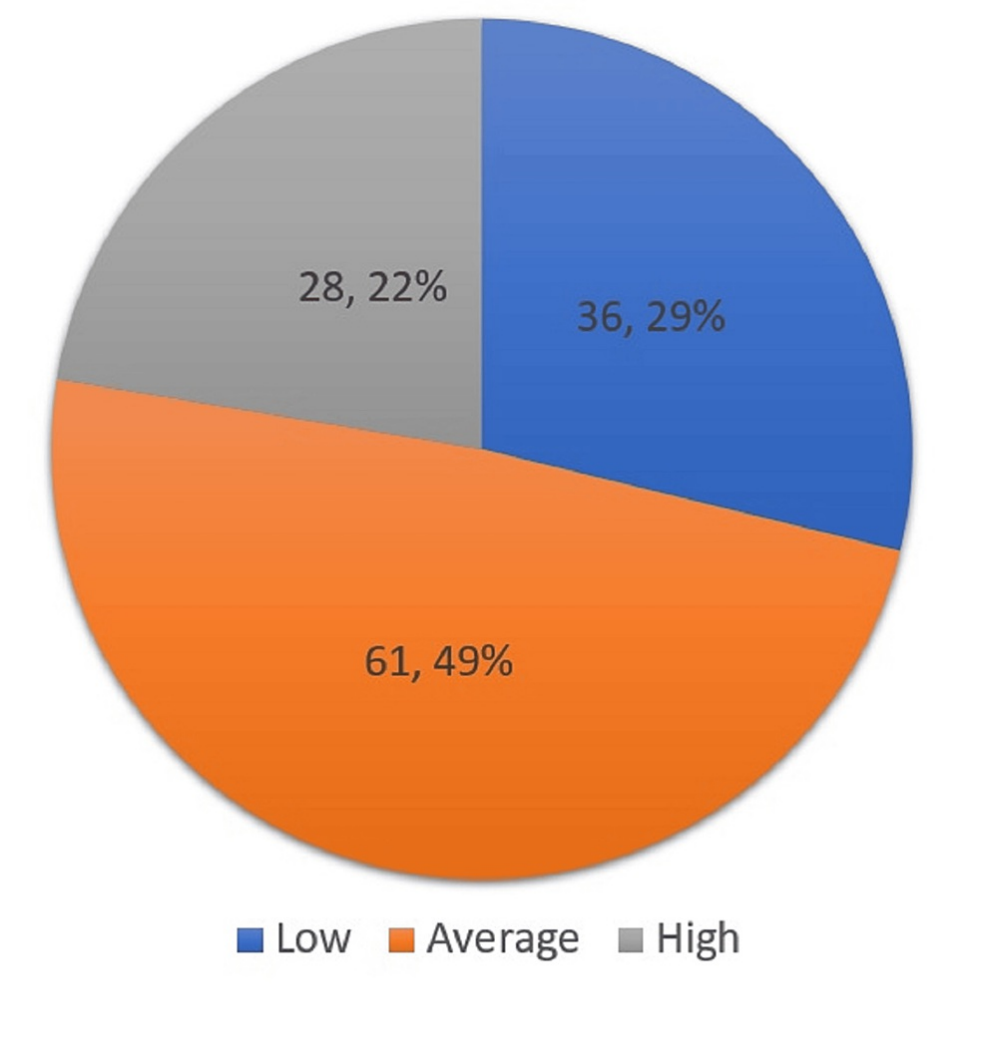
NICU nurses' knowledge level regarding neonatal pain assessment NICU= Neonatal Intensive Care Unit.

For the level of knowledge of neonatal pain assessment among the NICU nurses, nearly half of the participants had an average level of knowledge regarding neonatal pain assessment (n= 61, 49%), 36 participants had a low level of knowledge (29%), and 28 had a high level of knowledge (22%) (Figure 1).

The level of knowledge regarding neonatal pain assessment was higher among non-Saudi nurses (P≤ 0.05). There was no significant difference in knowledge between public and private hospital nurses. No significant relationship existed between other demographic variables and the nurses' knowledge regarding neonatal pain assessment (Table 3).

**Table 3:** Relationship between demographic characteristics and knowledge of nurses regarding neonatal pain ^*^ = P-value is considered significant; r= Pearson correlation coefficient; t= Independent t-test; F= One way analysis of variance (ANOVA)

Variable	Mean Level of Knowledge	Test	P-Value
Age	0.63	r= 0.14	0.12
Years of experience in neonatal nursing	0.63	r= 0.03	0.70
Marital status:			
Single	0.64	t= 0.16	0.88
Married	0.63		
Citizenship:			
Saudi	0.53	t= 5.20	0.001^*^
Non-Saudi	0.72		
Education:			
College diploma	0.62	F= 1	0.37
Bachelor	0.63		
Master and above	0.64		
Hospital Type:			
Public	0.63	t= 0.02	0.98
Private	0.63		

## Discussion

This study showed that 71% of NICU nurses had an average to high level of knowledge regarding neonatal pain assessment, and 29% of NICU nurses had inadequate knowledge, but it can be improved. Nurses demonstrated an understanding that minor procedures can contribute to pain in neonates and that prolonged pain in neonates can have adverse effects. This knowledge is crucial as newborns in the NICU are subjected to numerous painful procedures throughout their hospitalization. Both preterm and full-term neonates experience these painful procedures, which can lead to negative consequences. The results of the current study are consistent with the findings of Carlsen Misic and colleagues' study, which reported that approximately half of the neonatal nurses in Sweden thought that they had sufficient knowledge about neonatal pain assessment, with those who had specialized education showing higher levels of neonatal pain knowledge. Knowledge acquisition of neonatal pain can be attributed to various factors, including formal education, specialized training, clinical experience, and collaboration with other nursing professionals [1].

Furthermore, the present study reveals that nurses were aware of several key aspects of neonatal pain. They recognized that neonates can experience pain and that preterm neonates are particularly vulnerable to neurodevelopmental impairments resulting from repeated painful procedures. Nurses also acknowledged that neonates, especially preterm infants, exhibit heightened pain sensitivity compared to older children and adults. The understanding that neonatal pain can have long-term adverse effects aligns with the findings of Perry and colleagues, who emphasized that neonates undergo various painful procedures and surgeries during their hospitalizations. This heightened sensitivity to pain in neonates, particularly preterm infants, is attributed to factors such as alterations in central somatosensory functions and brain structure, as documented by [13]. The consequences of pain in neonates extend beyond immediate discomfort. Adverse effects include impaired responses later in life, such as poor neurodevelopment, emotional responses, and somatosensory processing [31-33].

In this study, NICU nurses showed a lack of knowledge regarding the validated pain assessment scales, especially the ones to be used for preterm neonates. However, these findings differ from those of Alburaey and colleagues, who conducted a study in the Eastern region of Saudi Arabia and reported adequate knowledge among NICU nurses regarding neonatal pain assessment and management [24]. Contrary to the findings of the present study, Wari and colleagues conducted a study in Ethiopia and reported adequate knowledge among nurses regarding neonatal pain assessment and management [31]. Similarly, Carlsen and colleagues found sufficient knowledge of pain assessment in newborns, with nurses regularly using pain assessment scales [1]. It is noteworthy to mention that nurses who got an education on neonatal pain assessment and management had higher knowledge regarding neonatal pain assessment than those who did not [1,31]. The disparity in results may be attributed to the more detailed and specific questions used in the present study, which included 18 items for neonatal pain assessment compared to previous studies. 

In interpreting the findings of this study, it is important to consider the variability in knowledge levels among NICU nurses regarding neonatal pain assessment. Identifying knowledge gaps highlights the need for targeted educational interventions and ongoing training programs to enhance nurses' understanding of neonatal pain and its assessment. Future research should explore the effectiveness of educational interventions in improving knowledge levels and the subsequent impact on pain management practices and patient outcomes. Additionally, efforts should be made to incorporate neonatal pain assessment training into nursing education curricula to ensure that healthcare professionals are equipped with the necessary knowledge and skills to provide optimal care for neonates. After adequate staff education, adopting one validated and age-appropriate pain assessment tool into policy and practice is also important, especially in participating institutions. 

This study used specific questions to assess the knowledge of nurses regarding neonatal pain assessment. These questions were constructed based on previous tools and literature, and they were validated by a panel of experts. However, this study acknowledges some limitations. The study was conducted in two hospital sectors (private and public) in Saudi Arabia and may not be generalizable to other settings or countries. Additionally, the study relied on self-report measures from the participating nurses, which may introduce potential biases or inaccuracies. Future research should consider larger sample sizes, multiple healthcare facilities, and a combination of objective and subjective measures to provide a more comprehensive understanding of neonatal pain assessment practices.

## Conclusions

Most NICU nurses had an average to a high level of knowledge regarding neonatal pain assessment. Yet, a significant proportion of NICU nurses had inadequate knowledge, which can be improved. Nurses need to be educated more and trained about neonatal pain assessment and the validated tools. There is also a need for NICUs in Saudi Arabia to adopt evidence-based practice recommendations for neonatal pain assessment, especially the validated pain assessment tools for both preterm and full-term neonates. Future research should recruit more samples from different health sectors and cover more NICUs in Saudi Arabia.
